# Detection and Characterization of Influenza A Virus Endemic Circulation in Suckling and Nursery Pigs Originating from Vaccinated Farms in the Same Production System

**DOI:** 10.3390/v16040626

**Published:** 2024-04-18

**Authors:** Alessandra Silva Dias, Amy L. Vincent Baker, Rodney B. Baker, Jianqiang Zhang, Michael A. Zeller, Pravina Kitikoon, Phillip C. Gauger

**Affiliations:** 1 Department of Preventive Veterinary Medicine, Minas Gerais State University, 6627 Antonio Carlos Avenue, Belo Horizonte 31620-295, MG, Brazil; alessandrasilvadias@yahoo.com.br; 2 Virus and Prion Research Unit, United States Department of Agriculture, National Animal Disease Center, Agricultural Research Service, 1920 Dayton Avenue, Ames, IA 50010, USA; amy.l.baker@usda.gov (A.L.V.B.); pravina.kitikoon@ceva.com (P.K.); 3 Department of Veterinary Diagnostic and Production Animal Medicine, Iowa State University, 1800 Christensen Drive, Ames, IA 50011, USA; rbbaker@iastate.edu (R.B.B.); jqzhang@iastate.edu (J.Z.); mazeller@iastate.edu (M.A.Z.); 4 Phillip Gauger of Veterinary Diagnostic and Production Animal Medicine, College of Veterinary Medicine, Iowa State University, 1800 Christensen Drive, Ames, IA 50011, USA

**Keywords:** swine, influenza A virus, endemic infection, sucking piglets, vaccine

## Abstract

Inactivated influenza A virus (IAV) vaccines help reduce clinical disease in suckling piglets, although endemic infections still exist. The objective of this study was to evaluate the detection of IAV in suckling and nursery piglets from IAV-vaccinated sows from farms with endemic IAV infections. Eight nasal swab collections were obtained from 135 two-week-old suckling piglets from four farms every other week from March to September 2013. Oral fluid samples were collected from the same group of nursery piglets. IAV RNA was detected in 1.64% and 31.01% of individual nasal swabs and oral fluids, respectively. H1N2 was detected most often, with sporadic detection of H1N1 and H3N2. Whole-genome sequences of IAV isolated from suckling piglets revealed an H1 hemagglutinin (HA) from the 1B.2.2.2 clade and N2 neuraminidase (NA) from the 2002A clade. The internal gene constellation of the endemic H1N2 was TTTTPT with a pandemic lineage matrix. The HA gene had 97.59% and 97.52% nucleotide and amino acid identities, respectively, to the H1 1B.2.2.2 used in the farm-specific vaccine. A similar H1 1B.2.2.2 was detected in the downstream nursery. These data demonstrate the low frequency of IAV detection in suckling piglets and downstream nurseries from farms with endemic infections in spite of using farm-specific IAV vaccines in sows.

## 1. Introduction

Influenza A virus (IAV) is one of the most important infectious agents frequently found in the porcine respiratory disease complex [1]. Clinical disease may vary from an absence of respiratory signs in pigs with subclinical endemic IAV to fever and severe respiratory signs during acute clinical infections [2]. Outbreaks of respiratory disease in pigs caused by IAV typically result in high morbidity, low mortality, and a sudden onset of clinical signs such as coughing, followed by the rapid recovery of the affected animals [3].

Classical H1N1 (cH1N1 or 1A lineage) was the prevalent genotype circulating in the United States (U.S.) swine population from the 1918 flu pandemic to 1998 [4], when a new H3N2 subtype containing swine (NP, M, and NS), avian (PB2 and PA) and human (HA, NA, and PB1) lineage internal genes were introduced into the swine population and designated as the triple-reassortant internal gene (TRIG) constellation [5]. Once established in swine, H3N2 evolved through reassortment with the endemic H1 clade 1A and the 1B clade of IAV that spilled over from humans in 2002 [4,6]. However, reassortment with the 2009 pandemic H1N1 (H1pdm09) and its internal genes, primarily the pandemic Matrix (pM), has led to a dramatic increase in the diversity of H3 IAV in swine, giving rise to the current clusters 3.1990.4, 3.1990.4.a, and 3.1990.4.b (U.S. clades IV, IVA, and IVB). Moreover, this has resulted in the establishment of different constellations of TRIG and pandemic internal gene lineages in both H1 and H3 viruses circulating in U.S. swine [7,8,9,10,11]. The increasing genetic diversity and complex relationship between IAVs circulating worldwide has given rise to three major global H1 lineages in swine (1A, 1B, and 1C) and several H3 IAV lineages (3.1990.4, 2010.1, and 2010.2) based on the year the virus spilled over from humans [7].

The vaccination of replacement gilts at farm entry and the mass vaccination of adult breeding swine on farms are important methods used to decrease IAV infection in piglets at weaning [12], contributing to the control of virus transmission within and between farms. Prior to 2012, all swine influenza vaccines approved in the U.S. were adjuvanted whole inactivated virus (WIV) vaccines [13]. However, the breadth of genetic diversity of contemporary IAV circulating in U.S. swine and potential mismatch with vaccine antigens has required the use of farm-specific vaccines to improve protection against the strains circulating at the farm level [13]. Animal health companies may require an IAV isolate to create WIV vaccines or may use sequences of the hemagglutinin (HA) gene for the production of replicon particle vaccines. Both platforms utilize multivalent products to increase cross-protection among different IAV subtypes, genetic clades, and strains [14,15,16,17]. This approach improves the ability to match vaccine antigens with endemic IAV that are specific to the farm and increases protection against infection [18,19,20,21,22].

Several strains of IAV have been reported to circulate simultaneously in swine farms, regardless of vaccination and in the absence of clinical disease [23,24]. The transportation of weaned piglets inadvertently infected with IAV is considered a major route of virus spread in North America [24,25,26,27]. The presence of susceptible neonatal or suckling piglets in breeding farms has been considered a mechanism for establishing endemic IAV infections [28], making this pig age one of the most important reservoirs and maintenance of subclinical IAV infections [22,24,25,29]. Despite the important role of suckling piglets in maintaining IAV circulation in swine-breeding farms, the prevalence of IAV associated with endemic infections remains low [12,25,30]. However, it is unclear whether the prevalence of IAV decreases over the production cycle or if IAV circulates continuously at subclinical levels [25], which complicates the ability to detect viruses from endemic infections through routine diagnostic sample types. The sporadic detection of endemic IAV may also affect the success of HA sequencing and virus isolation, which also decreases the ability to effectively control disease if farm-specific vaccines are unable to be produced.

The detection of endemic IAV in suckling and nursery piglets has become an important aspect of control, making it necessary to monitor the strains circulating in swine farms to properly enable the selection and use of an effective vaccine product. Currently, there are few studies reporting the presence of endemic IAV in suckling pigs located on breeding farms using farm-specific vaccines that include antigens matched to viruses circulating at the farm level and how that may impact the magnitude of IAV circulation in the nursery. In the present study, we evaluated the detection of IAV circulation in four breed-to-wean Midwestern U.S. swine farms from one production system using vaccines in the adult breeding herd. The specific objective was to evaluate the level of detection of IAV in the suckling piglet population post-vaccination with a farm-specific vaccine closely matched to strains circulating on the farm and its impact on the downstream pigs in the nursery.

## 2. Materials and Methods

### 2.1. Experimental Design and Sample Collection

This study was conducted with the approval of the Institutional Animal Care and Use Committee at Iowa State University as diagnostic investigation protocol number 2-13-7504-S.

Four swine-breeding farms (F1 to F4) containing 4200 to 6200 animals from the same production system located in the Midwestern U.S. with a history of IAV infection and using vaccines in the breeding herd were selected for this study (Table 1). Sows at all four farms were previously vaccinated with a commercial WIV product (MaxiVac Excell^®^ 5.0 Merck Animal Health) according to the manufacturer’s recommendation for at least one year prior to initiating the current study. The commercial vaccine lacked similar IAV antigens to the farm-specific strains based on genetic clade and sequencing. A multivalent, HA replicon-particle (HA-RP) farm-specific vaccine was produced with 3 HA antigens that were selected from IAV detected in grow-finish or adult swine from the production system in 2012. One week prior to sample collection, 4 March 2013, all adult breeding swine in F1, F2, and F3 were mass-vaccinated with a farm-specific HA-RP that contained H1 1B.2.2.2 (U.S. δ1b), H1 1A.3.3.3 (U.S. γ), and H3 3.1990.4.b (U.S. cluster IVB) antigens. A boost dose of HA-RP was administered on April 1 to F1 and F3 and on April 15 to F2. A single dose of the HA-RP vaccine was administered to F4 on 25 March 2013.

Samples were collected from all four farms every other week between March and May and July and August 2013 for a total of eight collections per farm (Table 1). The study was interrupted during the month of June due to an outbreak of porcine epidemic diarrhea virus (PEDV) and increased biosecurity on the study farms prohibiting project researchers from collecting samples. One hundred and thirty-five nasal swabs from 12- to 17-day-old piglets were collected by litter during each farm visit, for a total of 4320 samples (Table 2). Post-collection, polyester swabs were immediately immersed in minimum essential media (MEM, Gibco, Waltham, MA, USA) and maintained on ice during transit from the farm. During sample collection at the sow farm, clinical signs of respiratory disease were recorded based on clinical impressions. Three to eight oral fluid samples were collected by farm personnel from the same groups of piglets after transport to the nursery at approximately 4–5 weeks old for a total of 158 oral fluids (Table 2). Briefly, oral fluid samples were collected by suspending unbleached cotton rope in the area of the heat mat and within reach of the piglets for approximately 30 min, as previously described [31]. Oral fluid was extracted by manually squeezing the rope inside a plastic bag, and the fluid was transferred into a conical tube and frozen prior to shipment to the laboratory. Due to farm personnel collecting oral fluid samples, clinical signs of respiratory disease were not recorded during sample collection, and sample numbers varied from different nurseries (Table 2). Nasal swabs and oral fluid samples were stored at −80 °C at the laboratory prior to analysis.

### 2.2. Influenza A Virus RNA Detection and Subtyping

Nasal swabs from each collection were pooled in groups of three for RNA extraction. Each pool of nasal swabs from suckling piglets and oral fluid from nursery pigs were extracted using a commercial MagMAX^TM^ Viral RNA Isolation Kit (Thermo Fisher Scientific, Waltham, MA, USA) according to the kit recommendations. Reverse transcription real-time PCR (RT-rtPCR) was performed using the Vet MAX^TM^-Gold SIV Detection Kit (Thermo Fisher Scientific) to evaluate the presence of IAV RNA according to the manufacturer’s recommendations. Samples with cycle threshold (Ct) values < 38.0 were considered positive.

Subtype-specific PCR (H1, H3, N1, N2) was conducted on all individual nasal swabs from RT-rtPCR-positive pools and on positive oral fluids using the commercial Vet MAX^TM^- SIV Subtyping RNA assay (Thermo Fisher Scientific) according to the kit’s instructions. Samples with Ct values < 35.0 were considered positive; values of 35.0 < Ct ≤ 38.0 were considered suspect; and values > 38.0 were considered negative.

### 2.3. Detection of Pandemic or North American TRIG Matrix Genes

Individual nasal swabs and oral fluid samples positive for IAV in the screening PCR were analyzed for the pandemic Matrix (pM) or the North American TRIG Matrix (TrM) genes using a differential RT-rtPCR assay, as previously described [32]. Samples with Ct values ≤ 40.0 were considered positive.

### 2.4. Influenza A Virus Isolation

Positive IAV RT-rtPCR nasal swabs were submitted for virus isolation in 48-well plates with confluent monolayers of Madin–Darby Canine Kidney (MDCK) cells using methods previously described [33]. Virus isolation was performed on IAV RT-rtPCR positive oral fluids using 25 cm^2^ cell culture flasks with MDCK confluent cell monolayers. Oral fluids were centrifuged at 1500× *g* for 10 min at 4 °C, and 300 µL of supernatant was used to inoculate the flasks previously rinsed three times with phosphate-buffered saline (PBS; pH 7.4; Gibco, Waltham, MA, USA). Flasks were incubated at 37 °C with 5% CO_2_ for one hour, after which, the inoculum was removed, and flasks were rinsed three times with PBS. Finally, MEM supplemented with 100 IU/mL of penicillin, 100 µg/mL of streptomycin, 0.4% fetal bovine serum, and 2 µg/mL of TPCK-treated trypsin was added, and cell cultures were incubated for up to seven days. Development of the cytopathic effect (CPE) was monitored, and the hemagglutination activity of the cell lysate was analyzed to verify virus isolation results. If negative at the first passage, cell cultures were subject to a second passage prior to reporting a negative virus isolation result. Positive supernatants upon virus isolation were stored at −80 °C.

### 2.5. Influenza A Virus Complete Genome Sequencing

Two H1N2 isolates from nasal swabs with the highest hemagglutination titer and three nasal swabs with RT-rtPCR Ct values ≤ 32 in the subtyping assay were selected for whole-genome sequencing (WGS). An H1N1- and H3N2 RT-rtPCR-positive oral fluid samples with Ct ≤ 30 in the subtyping RT-rtPCR were also selected for WGS. The IAV WGS was conducted with an Ion Personal Genome Machine^TM^ (PGM^TM^) System platform, as previously described [34,35]. The Sanger method was used to sequence the HA gene from oral fluid samples if WGS attempts were unsuccessful. Briefly, viral RNA was extracted using the MagMAX^TM^ Viral RNA Isolation Kit (Thermo Fisher Scientific, Waltham, MA, USA), as described for RT-rtPCR. Reverse transcription PCR (RT-PCR) amplified the HA using 1 µL each of the forward and reverse primers targeting conserved regions of the HA and 12.5 µL of 2x qScript^TM^ XLT 1-Step RT-qPCR ToughMix (Quantabio, Beverly, MA, USA), 1 µL of 25x qScript XLT 1-Step RT-PCR (Quantabio), 5.5 µL of nuclease-free water, and 4 µL of template for a 25 µL reaction, per ISU VDL standard operating procedures. One positive extraction control, one negative extraction control, and one negative amplification control were included with the reaction. The RT-PCR was performed using an ABI 2720 thermal cycler with the following cycling conditions: 1 cycle at 48 °C for 20 min; 1 cycle at 94 °C for 3 min; 45 cycles at 94 °C for 30 s, 55 °C for 50 s, and 68 °C for 2 min; and 1 cycle at 68 °C for 7 min. The RT-PCR product was purified using the ExoSAP-IT^TM^ PCR Product Cleanup Reagent (Thermo Fisher Scientific) per manufacturer instructions. Samples were submitted to the Iowa State University DNA facility for nucleotide sequencing. The Lasergene software v11.2 (DNAStar, Madison, WI, USA) was used to compile sequences.

### 2.6. Influenza A Virus Phylogenetic Analysis

The H1 and H3 HA and N1 and N2 -NA sequences were aligned using MAFTT v7.490 [36]. For each alignment, the maximum likelihood tree was inferred by IQ-TREE v1.6.12 [37] using a general time reversible model of nucleotide substitution.

## 3. Results

### 3.1. Influenza A Virus Respiratory Clinical Signs

Clinical signs typical of IAV respiratory disease were only observed at F2 during the first week of sample collection (11–15 March). A deep, barking cough was observed throughout the farrowing room, affecting approximately 20% of dams and 10% of suckling piglets at 2–3 weeks old. Clinical signs associated with IAV were not observed on subsequent visits to F2 and were not apparent at F1, F3, or F4 throughout the entire sampling period. Oral fluid samples were collected from nursery pigs by farm personnel, and clinical signs of nursery pigs were not recorded.

### 3.2. Detection of Influenza A Virus RNA in Suckling Pig Nasal Swabs

Influenza A virus RNA was detected using a screening RT-rtPCR in 2.22% of the pooled nasal swabs (32/1440 pooled nasal swabs) across all farms and sample times). A majority of IAV RT-rtPCR-positive pools from nasal swabs were detected at Farm 2 at the first (40.00%; 18/45 pools) and second (15.56%; 7/45 pools) sample collections, as expected based on clinical impressions. IAV RNA was detected only sporadically in pooled nasal swabs from F1, F3, and F4 throughout the study. The RT-rtPCR Ct values ranged from 21.1 to 37.8 and were lowest at F2 on the March 11–15 sampling, when respiratory clinical signs were also observed. However, RT-rtPCR Ct values ranged from 31.2 to 37.8 during subsequent sample times across all farms.

Influenza A virus subtyping was performed to detect the presence of H1, H3, N1, or N2 in individual nasal swabs from the IAV RT-rtPCR-positive pooled samples. The assay detected the presence of H1, H3, and mixed H1/H3 subtypes in 1.64% (71/4320) of the individual nasal swabs based on the RT-rtPCR detection of the HA gene (Table 3). An additional 1.11% (48/4320) of the nasal swabs demonstrated Ct values in the suspect range (Ct 35–37.9) for H1, H3, or both subtypes (Table 3). Collectively, there were 57 H1, 3 H3, and 11 mixed H1-H3 infections. At minimum, 1.76% (76/4320) of the nasal swabs were RT-rtPCR-positive for N2 NA (Table 4). In addition, at least 16 nasal swabs demonstrated N1 NA detected at suspect Ct values at F2 in the first two samplings, suggesting the presence of an additional NA subtype and a potential mixed subtype infection at subclinical levels (Table 4).

The largest number of H1/H3 mixed infections was detected in week 1 (11–15 March) at F2, corresponding with clinical influenza illness observed in the farrowing room in sows and piglets. The virus, regardless of subtype, was not detected in nasal swabs from F2 during the final six collections, suggesting potential HA- RP vaccine efficacy (Table 3). In addition, H1 and N2 were the only HA and NA subtypes detected in F1, F3, and F4, except for a suspect H3 detected in F4 and F1 during week 6 (8–12 July) and week 7 (22–26 July), respectively.

### 3.3. Detection of Influenza A Virus RNA in Nursery Pig Oral Fluids

Oral fluid samples were collected by farm personnel and varied in quantity and volume between farms. A total of 158 samples were collected, and 31.01% (49/158) were RT-rtPCR-positive for IAV. Similar to the nasal swab results, Farm 2 demonstrated the highest number of RT-rtPCR-positive oral fluids at 51.02% (25/49). Farms 1, 3, and 4 demonstrated 14.28%, 10.20%, and 24.49% RT-rtPCR-positive oral fluids, respectively. Among the RT-rtPCR-positive samples, 28.57% were H1 (14/49), and 6.12% were H3 (3/49) (Table 5). Approximately 36.73% (18/49) of the subtyping RT-rtPCR-positive oral fluids were considered suspect (12 H1, 3 H3, and 3 mixed H1/H3 infections; Table 5), and 28.57% (14/49) did not subtype with either H1 or H3.

In total,18.37% (9/49) of the oral fluids were RT-rtPCR-positive for only the N2 subtype and 36.73% (18/49) for N2 were detected at the suspect level and corresponded to a suspect or negative H1 or H3 subtype, indicating the detection of low levels of N2 in spite of negative H1 or H3 detection. The RT-rtPCR-positive samples in the IAV screening PCR assays from F1 and F3 were too weak to detect a positive subtype.

Only 18.37% (9/49) of the screening RT-rtPCR-positive oral fluid samples had Ct values ≤ 30.0, suggesting that a majority of the positive samples were detected as potential subclinical infections with limited quantities of virus present at the time of sample collection. In addition, F2 had H1 and H3 subtypes detected in nasal swabs, while H3 was not detected in oral fluids (Table 3 and Table 5). In contrast, F4 had the H1N2 subtype detected in nasal swabs and one H3 detected at suspect levels, whereas only H3N2 was detected in oral fluids, with only suspect levels of H1 after pigs were transported to the nursery.

### 3.4. Detection of Pandemic or North American TRIG Matrix

A total of 32.89% (25/76) of the H1, H3, and N2 subtype-positive nasal swabs demonstrated the pM, all from F2. The North American TRIG M was not detected in nasal swabs. Some subtyping RT-rtPCR-positive nasal swabs were negative for either M gene lineage (pdm or TRIG), possibly due to weak Ct values and low concentrations of virus in the sample. For oral fluids, 51.02% (25/49) of RT-rtPCR-screening-positive samples demonstrated the pM gene, detected in F2 and F4. Three (6.12%) oral fluids had the North American TRIG M gene and were detected exclusively at F4 (April 8–12 sampling), suggesting the co-circulation of different internal gene constellations and potential for reassortment with strains that contained the pM. Approximately 42.89% (21/49) of RT-rtPCR-positive oral fluid samples were negative for both the pM and TRIG M genes, likely due to weak Ct values and low concentrations of the target in the sample.

### 3.5. Influenza A Virus Isolation

In total, 14.58% (14/96) of the nasal swabs were positive for virus isolation. The isolates were H1N2 from F2 during the 25–29 March collection. Virus isolation was not successful from F1, F3, and F4. All RT-rtPCR-positive oral fluids were negative for virus isolation.

### 3.6. Influenza A Virus Gene Sequencing

**Figure 1:**
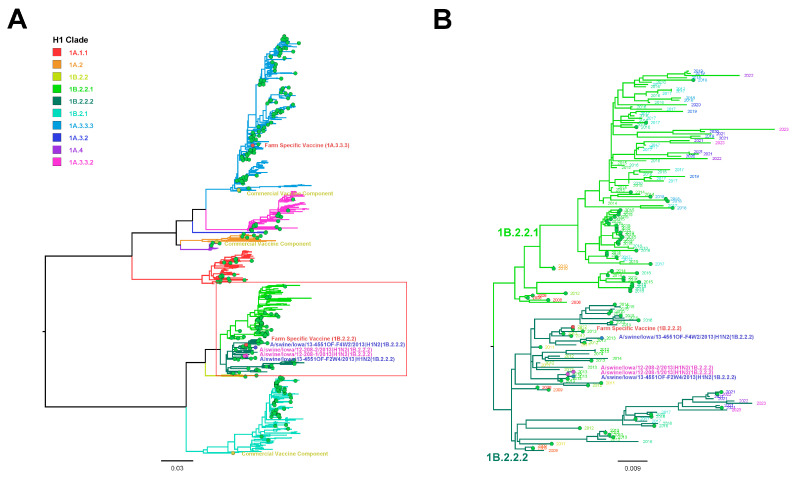
(**A**) Maximum likelihood phylogenetic tree of H1 hemagglutinin genes, including 2 H1 1B.2.2.2 (δ1b; magenta) sequences reported in this study from suckling pigs, 2 H1 1B.2.2.2 sequences from F2 and F4 (δ1b; blue) reported in this study from nursery pigs, farm-specific vaccine antigen sequences used during the study period (red), commercial vaccine antigen sequences (MaxiVac Excell^®^ 5; yellow), H1 IAV detected in 2012–2023 across the production system (green), and reference sequences representing IAV clades circulating in swine (branches without circles). (**B**) Maximum likelihood phylogenetic tree of H1 1B clade of hemagglutinin sequences represented in the red box in (**A**). Taxa are indicated by year of detection.

Five H1N2 samples were submitted for WGS; two IAV isolates from F2; and one nasal swab sample each from F1, F2, and F3. Only the two IAV isolates from F2 had all eight complete gene segments sequenced, as WGS failed on individual nasal swab samples collected from F1, F2, and F3. Phylogenetic analysis indicated F2 HA was the H1 1B.2.2.2 (U.S. clade δ1b) cluster with a 2002A N2 and pM gene consistent with the Matrix RT-rtPCR results. Moreover, the PB2, PB1, PA, NS, and NP segments were from the North American TRIG. H1 1B.2.2.2 F2 HA was 97.59% and 97.52% similar to the nucleotide and amino acid sequences, respectively, of the H1 1B.2.2.2 present in the farm-specific vaccine. The commercial vaccine used in the production system prior to the HA-RP vaccine did not contain the H1 1B cluster IAV. Two oral fluid samples were sequenced using Sanger methods and generated H1 HA sequences from F2 at week 4 and F4 at week 2. The H1 detected in these nursery pig samples was also H1 1B.2.2.2, which ranged from 99.62% to 99.88% nucleotide homology to both F2 sequences detected in the suckling pigs, indicating an epidemiologic relationship between infected suckling pigs and downstream nursery swine at F2 and F4. The phylogenetic relationship between the H1 IAV F2 HA sequences detected in nasal swabs and oral fluids; the commercial and farm-specific H1 vaccine antigens; H1 HA detected in the production system from 2012 to 2023; and H1 HA reference strains are presented in Figure 1A. The H1 1B clade, including the phylogenetic relationship between the H1 F2 HA sequences representing nasal swabs and oral fluids; the farm-specific H1 vaccine antigens; H1 1B HA detected in the production system from 2012to 2023; and H1 1B HA reference strains are presented in Figure 1B.

**Figure 2:**
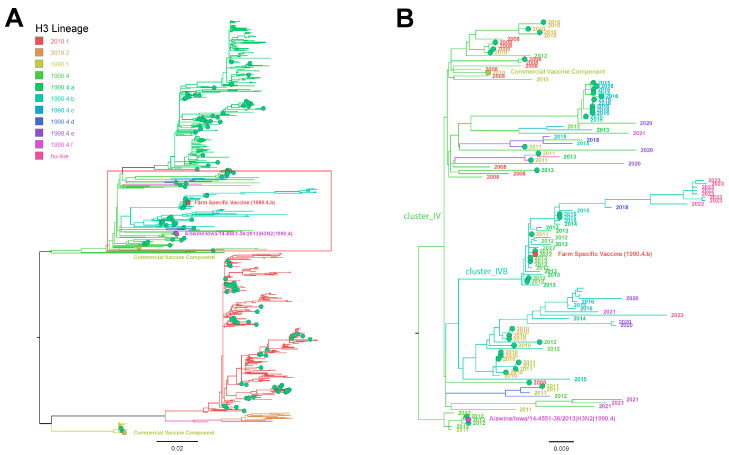
(**A**) Maximum likelihood phylogenetic tree of H3 hemagglutinin genes including 1 H3 3.1990.4 (cluster IV; magenta) sequence reported in this study from nursery pigs, the farm-specific vaccine antigen sequence used during the study period (red), commercial vaccine antigen sequences (MaxiVac Excell^®^ 5; yellow), H3 IAV detected in 2012–2023 across the production system (green), and reference sequences representing H3 IAV clades circulating in swine (branches without circles). (**B**) Maximum likelihood phylogenetic tree of H3 3.1990.4 clade of hemagglutinin sequences represented in the red box in (**A**). Taxa are indicated by year of detection.

Two H3N2 oral fluid samples from F4 and three from F2, based on subtyping RT-rtPCR, were selected for WGS but failed to generate sequences. Sanger sequencing was used to generate an H3 HA sequence from oral fluids from F4 at week 3. Although H3 sequences were not generated from nasal swabs for comparison, H3 HA from F4 oral fluid was designated clade 3.1990.4, and the H3 HA-RP vaccine strain was designated clade 3.1990.4.b, indicating at least one of the H3s circulating in nursery pigs was from a different phylogenetic clade compared with the vaccine antigen and represented only 94.70% and 94.88% nucleotide and amino acid homologies, respectively. The phylogenetic relationship between the F4 H3 oral fluid HA sequence, the commercial and farm-specific H3 vaccine antigens, H3 HA detected in the production system from 2012 to 2023, and H3 HA reference strains are presented in Figure 2A. The phylogenetic relationship between the F4 H3 3.1990.4 HA oral fluid sequence, the farm-specific H3 3.1990.4.b vaccine antigen, H3 3.1990.4 HA detected in the production system from 2012 to 2023, and the H3 3.1990.4 reference strains are presented in Figure 2B.

N2 NA sequenced from nasal swabs detected at F2 and in the nursery pig oral fluids from F2 and F4, regardless of pairing to H1 or H3, were the 2002A clade. Sequencing was not successful for N1 detected at suspect levels in nasal swabs from F2. A comparison of oral fluid N2 sequences from F2 and F4 to the N2 sequence from F2 nasal swabs ranged in nucleotide homology from 98.44% to 99.79%. Nucleotide comparison between only oral fluid N2 ranged from 98.16% to 98.51%. These data suggest that the endemic N2 detected at F2 may also have been circulating in the downstream nursery pigs at F2 and F4. It remains unknown if the low detection of IAV in the suckling and nursery pigs from F1 and F3 was similar to circulating strains detected at F2 and F4 due to the inability to perform sequencing.

## 4. Discussion

There are few studies available that report the presence of endemic IAV in suckling piglets located on breeding farms using farm-specific vaccines that include antigens matched to viruses circulating at the farm level and its impact on virus detection in the nursery. This study is significant in that it highlights the low frequency of detection of IAV infection in suckling piglets in spite of using farm-specific vaccines and the presence of similar IAV circulating in downstream nursery pigs from four different swine farms in the same production system located in the Midwestern U.S. These data demonstrate the sporadic detection of H1, H3, N1, and N2 IAV in approximately 1.64% of nasal swabs from suckling piglets across all farms during the study (Table 3), which confirms the presence of endemic IAV circulating at low prevalence with variation observed between farms (0.1–4.5%) and sample collection time (March–August 2013). These data suggest individual farms may vary in terms of IAV infection and health status even within the same production system and also emphasize the importance of using the appropriate sample size and collection frequency to ensure adequate surveillance of IAV in these populations.

Farm 2 had a higher percentage of H1- and H3-positive nasal swabs (5.6% [60/1080]; Table 3) detected during the first two sample collections. This is consistent with the higher N1 and N2 detection (Table 4) in the same F2 piglet population, as well as the higher frequency of detection in oral fluids collected from the same F2 nursery pigs (Table 5). This suggests that IAV infection in the suckling pig population, even when dams are vaccinated for IAV, may influence the presence of the virus in recently weaned pigs. Farm 2 demonstrated clinical signs consistent with IAV infection over the first and second weeks of sample collection, which coincided with the only IAV-positive samples detected on that farm. The HA-RP vaccine administered to the breeding animals included an H1 1B.2.2.2 sequence that was similar to the IAV circulating on the farm, suggesting the vaccine induced immune response may have reduced clinical signs and IAV detection during the subsequent 4 months of the study. However, it remains unknown if the virus would have been detected on F2 after the month of August when sample collection ended or into the fall season when IAV is more common in swine due to the seasonality of virus circulation [11,38,39].

As expected, nasal swabs from F2 had lower RT-rtPCR Ct values, which likely influenced the success of virus isolation and WGS from samples collected during the first 2 weeks of the study. However, sporadic detection of H1 and H3 IAV, often at suspect Ct values based on RT-rtPCR, occurred on F1, F3, and F4, supporting the conclusion that low levels of endemic virus circulated. Unfortunately, endemic influenza infections in suckling pigs are often detected at low concentrations of virus regardless of the sample type, which, in this study, precluded the ability to sequence additional samples and confirm how similar the IAV was across all four farms. These data demonstrate the unique viral dynamics present at different swine farms, which prohibits the assumption that the virus strain and prevalence are similar within a production system among different breeding farms and may require the implementation of farm-level vaccination strategies to effectively control the virus.

Although the data from this IAV study were collected in 2013, the results remain consistent with reports of endemic IAV detected in suckling piglets from contemporary swine production systems in the U.S. [22,25,29,40]. Endemic IAV in the preweaning pig population on breeding farms continues to play an important role in maintaining the virus in swine in the U.S. [24,25,39,41,42]. However, these infections may persist undetected or lack detection if the piglet population is not included in routine surveillance or if the sample size is incompatible with the expected prevalence [43]. In the current study, 135 nasal swabs collected from suckling piglets using bi-weekly collection are a sample size and collection frequency capable of detecting <1% prevalence of infection. When prevalence is low, insufficient sample sizes may impede the ability to detect IAV, which may also occur on vaccinated farms and is what may have been demonstrated in this study. However, contemporary sample collection methods and sample types used for IAV detection have improved since the time of this study and include the use of population sample types. Udder wipes and family oral fluids (oral fluids representing the dam and her litter) are currently used more often on breeding farms and represent detection at the litter level. These sample types have the advantages of (1) representing more piglets in the sample, (2) increasing the opportunity to detect the low prevalence of a pathogen, and (3) reducing the cost of diagnostic testing [44,45]. In addition, udder wipes have proven useful for sequencing and virus isolation, which helps support the use of population sample types for monitoring IAV in suckling pigs.

Prior studies have demonstrated the efficacy of using farm-specific vaccines that contain IAV antigens genetically similar to what is circulating on breeding farms, which may also reduce virus detection to comparable levels, as observed in F2 in the current study [12,18,20,21,30,46]. The sporadic and low detection of IAV in nasal swabs from the suckling pigs on F1, F3, and F4 (Table 3 and Table 4) may also suggest the potential efficacy of the HA-RP vaccine. Unfortunately, HA gene sequences could not be generated from the low amounts of virus in nasal swabs collected during the study period from F1, F3, and F4 to confirm the similarity of vaccine antigens and their circulating strains. In addition, the prevalence of virus circulation prior to vaccination was unknown on all farms, prohibiting an accurate assessment of HA-RP vaccine efficacy.

Evaluating antibody levels in breeding animals and maternally derived antibodies (MDAs) in suckling piglets was beyond the scope of this report. However, it is expected that MDAs induced by the HA-RP vaccine will eventually protect piglets against clinical disease on F2 and may have been responsible for the low level of virus detection on all farms, as described in prior similar studies [18,19,20,21], depending on the age at the time of infection, the number of circulating MDAs, and MDA cross-protection with the strains circulating on the farm [20]. However, despite the apparent impact of the HA-RP vaccine on IAV detection in suckling and nursery pigs following the vaccination of the sows during the study period, endemic IAV continued to circulate and be detected over time. This is demonstrated by recent IAV sequences (2012–2023) that are included from this production system in Figure 1 and Figure 2, suggesting that vaccine antigens may need updating on a routine basis, supporting the need for consistent surveillance at the farm level. In this study, sows were vaccinated with a commercial product for at least one year prior to changing to a farm-specific HA-RP vaccine at the start of sample collection. Historically, only WIV commercial vaccines with adjuvants were available for use to control IAV prior to 2012 [13]. As a consequence of the rapidly increasing IAV genetic diversity, commercial vaccine antigens can quickly become outdated and mismatched to circulating strains at the genetic clade or nucleotide level [13,47]. It became more common to use farm-specific vaccines to increase the probability of matching vaccine antigens and circulating strains and to allow more timely vaccine antigen updates to occur [17,30,39].

Oral fluid is a common sample type for the surveillance of IAV in swine and, in this study, provided a genetic comparison of viruses between suckling and nursery pigs. RT-rtPCR detected 31.01% (49/158) of the oral fluids positive for IAV RNA, and similar to detection in the nasal swabs, most of the positive nursery pigs were from F2 at the first and second sample collections, although the F1, F3, and F4 nursery pigs had sporadic positive or suspect levels of IAV RNA. Corroborating other studies, our results suggest that endemic IAV in suckling piglets plays an important role in disseminating viruses at weaning to epidemiologically connected nurseries where piglets are often mixed by site, barn, or room [24,25,30,48]. Although clinical signs were not recorded, as nursery pigs age, the level of MDAs declines at variable rates, increasing the susceptibility of pigs to IAV infection and respiratory disease [49]. This may suggest why detection rates were higher in the nursery pigs compared with the endemic infections detected on the breeding farms.

Although oral fluids are a common surveillance sample for IAV in swine due to their ease of collection and the ability to represent populations of pigs [50], virus isolation from this sample type is difficult [51,52]. Fortunately, some IAV vaccine platforms, such as the HA-RP vaccine used on this farm, do not require virus isolates for vaccine production and need only the HA gene sequence [15,53,54]. Unfortunately, H3 HA sequences were not generated from suckling pigs in spite of detection (Table 3), although the H3 3.1990.4 sequence generated in F4 nursery pigs did not match the same clade of the IAV antigen that was used in the HA-RP vaccine (3.1990.4.b), suggesting that a vaccine antigen update may be necessary (Figure 2). The H1 and H3 sequences used as the HA-RP antigen were detected in adult swine in 2012. However, the vaccine was not implemented until March of 2013. These data suggest circulating viruses may genetically change from the time of antigen or strain selection and when farm-specific vaccines are produced, which, depending on multiple factors, may require several months. Interestingly, the H1 1B.2.2.2 sequence detected at F2 was 97.5% similar to the nucleotide sequence of the H1 1B vaccine antigen, suggesting potential genetic drift in progress (Figure 1). These data highlight the challenges associated with selecting vaccine antigens for genetically diverse viruses like IAV, even when originating from the farm of origin.

Influenza A virus is capable of reassorting its internal gene segments during replication as one method of increasing IAV genetic diversity [4]. Although reassortment has always been a feature of IAV, the rate of occurrence may have been underestimated until WGS methods became more accessible, which enabled the detection of HA and NA genes in combination with internal gene segments. The emergence of H1pdm09 in humans and its spillover into swine appears to have accelerated the rate of viral reassortment with endemic IAV in pigs [38,55,56]. Indeed, IAV surveillance data have demonstrated a marked increase in the presence of the pM gene at approximately the time this study was conducted, and the pM from H1pdm09 has now replaced the TRIG lineage M gene entirely in the IAV circulating in U.S. swine [57,58]. In our study, 32.89% (25/76) of RT-rtPCR-subtype-positive nasal swabs also demonstrated the pM gene, all from F2. In contrast, 51.02% (25/49) of the oral fluid samples, all from F2 and F4, had the pM gene, and 6.12% (3/49) of oral fluids retained the TRIG M gene. However, the pigs at F4 also had IAV circulating with the pM gene in suckling piglets and IAV with either pM or TRIG M in weaned pigs based on the RT-rtPCR. These data suggest that viruses with different internal gene constellations likely co-circulated in the pigs and increased the opportunity for reassortment, which ultimately may have contributed to increasing the genetic diversity of IAV at the farm level. Reassortant swine IAV that acquired the pM have also been detected as variant human infections based on previous reports and provides an example of the potential bidirectional transmission of IAV between humans and swine and apparent public health risks [59,60].

## 5. Conclusions

In summary, this study demonstrates the detection of different subtypes and genetic clades of IAV co-circulating as endemic infections in suckling pigs and its impact on the detection of similar IAV in the same nursery pig populations. These data suggest that endemically infected piglets on breeding farms may be a reservoir for one or more strains of influenza virus in spite of the use of vaccines as a measure of control. Also, suckling piglets may be a source of infection for piglets when moving into the nursery, which may impact the health of other farms within the production system. These data also emphasize the importance of the IAV surveillance of swine breeding herds and the potential use of farm-specific vaccines, which may benefit matching the vaccine antigens with currently circulating strains. However, more studies are needed to improve knowledge regarding vaccine efficacy and its ability to provide more consistent and cross-protective immunity to reduce the prevalence of endemic influenza infections in suckling piglets.

## Figures and Tables

**Table 1:** Sample collection timeline and farm-specific whole-herd vaccine administration dates for F1, F2, F3, and F4 in 2013.

	Sample Collection							
	1	2	3	4	5	6	7	8
Collection Date	11–15 March	25–29 March	8–12 April	22–26 April	6–9 May	8–12 July	22–26 July	5–9 August
Vaccine Dose	Prime dose	Prime dose	Boost dose	Boost dose				
Administration Date	March 4	March 25	April 1	April 15				
Farms Vaccinated	F1, F2, F3	F4 *	F1, F3	F2				

* F4 did not receive a boost dose of the farm-specific vaccine after the whole-herd prime dose.

**Table 2:** Experimental design.

Group	Location	Age (Days)	Sample Type	Samples/Farm	# of Farms	# of Samplings	Total Samples
Suckling Pigs	Farrowing	12–17	Nasal Swabs	135	4	8	4320
Nursery Pigs	Nursery	28–35	Oral Fluids	3–8	4	8	158

**Table 3:** Influenza A virus H1 and H3 RT-rtPCR-positive nasal swabs by farm during bi-weekly sample collection with mean positive cycle threshold values and standard error of the mean.

	Farm 1					Farm 2					Farm 3					Farm 4				
	H1		H3		H1/H3	H1		H3		H1/H3	H1		H3		H1/H3	H1		H3		H1/H3
Time	#/# Pos. (%)	Mean Ct [SEM]	#/# Pos. (%)	Mean Ct [SEM]	#/# Pos. (%)	#/# Pos. (%)	Mean Ct [SEM]	#/# Pos. (%)	Mean Ct [SEM]	#/# Pos. (%)	#/# Pos. (%)	Mean Ct [SEM]	#/# Pos. (%)	Mean Ct [SEM]	#/# Pos. (%)	#/# Pos. (%)	Mean Ct [SEM]	#/# Pos. (%)	Mean Ct [SEM]	#/# Pos. (%)
1	-	-	-	-	-	38/135 (28.1) 5 *	29.6 [0.6]	3/135 (2.2) 29 *	34.4 [0.4]	11/135 (8.1) 2 *	1/135 (0.7) 1 *	31.7 NA	-	-	-	2/135 (1.5)	33.8 [1.1]	-	-	-
2	6/135 (4.4)	32.6 [0.6]	-	-	-	8/135 (6.0) 6 *	33.3 [0.6]	-	-	-	-	-	-	-	-	-	-	-	-	-
3	-	-	-	-	-	-	-	-	-	-	-	-	-	-	-	-	-	-	-	-
4	-	-	-	-	-	-	-	-	-	-	-	-	-	-	-	-	-	-	-	-
5	-	-	-	-	-	-	-	-	-	-	-	-	-	-	-	-	-	-	-	-
6	2/135 (1.5) 1 *	33.3 [0.1]	-	-	-	-	-	-	-	-	-	-	-	-	-	-	-	1 *	NA	-
7	2 *	NA	1 *	NA	-	-	-	-	-	-	-	-	-	-	-	-	-	-	-	-
8	-	-	-	-	-	-	-	-	-	-	-	-	-	-	-	-	-	-	-	-
Pos/ Farm	8/1080 (0.7)	32.8 [0.4]	-	-	-	46/1080 (4.2)	30.2 [0.5]	3/1080 (0.3)	34.4 [0.8]	11/1080 (1.0)	1/1080 (0.1)	31.7 NA	-	-	-	2/1080 (0.2)	33.8 [1.1]	-	-	-

H1/H3: number of positive mixed infections or partial subtypes; #/#: number of H1 or H3 subtypes/total samples tested; ( ): percentage of H1- or H3-positive nasal swabs per month; [ ]: standard error of the mean [SEM]; *: samples with suspect Ct values (35.0 < Ct ≤ 38.0); NA: not applicable; -: negative (Ct > 38.0).

**Table 4:** Influenza A virus N1 and N2 RT-rtPCR-positive nasal swabs by farm during bi-weekly sample collection with mean positive cycle threshold values and standard error of the mean.

	Farm 1					Farm 2					Farm 3					Farm 4				
	N1		N2		N1/N2	N1		N2		N1/N2	N1		N2		N1/N2	N1		N2		N1/N2
Time	#/# Pos. (%)	Mean Ct [SEM]	#/# Pos. (%)	Mean Ct [SEM]	#/# Pos. (%)	#/# Pos. (%)	Mean Ct [SEM]	#/# Pos. (%)	Mean Ct [SEM]	#/# Pos. (%)	#/# Pos. (%)	Mean Ct [SEM]	#/# Pos. (%)	Mean Ct [SEM]	#/# Pos. (%)	#/# Pos. (%)	Mean Ct [SEM]	#/# Pos. (%)	Mean Ct [SEM]	#/# Pos. (%)
1	-	-	-	-	-	15 *	NA	54/135 (28.1)	29.7 [0.5]	-	-	-	2/135 (1.5)	32.8 [1.9]	-	-	-	2/135 (1.5)	33.4 [0.9]	-
2	-	-	6/135 (4.4)	32.0 [0.5]	-	1 *	NA	10/135 (7.4) 6 *	32.9 [0.4]	-	-	-	-	-	-	-	-	-	-	-
3	-	-	-	-	-	-	-	-	-	-	-	-	-	-	-	-	-	-	-	-
4	-	-	-	-	-	-	-	-	-	-	-	-	-	-	-	-	-	-	-	-
5	-	-	-	-	-	-	-	-	-	-	-	-	-	-	-	-	-	-	-	-
6	-	-	2/135 (1.5) 1 *	32.9 [0.5]	-	-	-	-	-	-	-	-	-	-	-	-	-	-	-	-
7	-	-	-	-	-	-	-	-	-	-	-	-	-	-	-	-	-	-	-	-
8	-	-	-	-	-	-	-	-	-	-	-	-	-	-	-	-	-	-	-	-
Pos/ Farm	-	-	8/1080 (0.7)	30.2 [0.4]	-	-	-	64/1080 (5.9)	30.2 [0.4]	-	-	-	2/1080 (0.2)	32.8 [1.9]	-	-	-	2/1080 (0.2)	33.4 [0.9]	-

H1/H3: number of positive mixed infections or partial subtypes; #/#: number of N1 or N2 subtypes/total samples tested; ( ): percentage of N1- or N2-positive nasal swabs per month; [ ]: standard error of the mean [SEM]; *: samples with suspect Ct values (35.0 < Ct ≤ 38.0); NA: not applicable; -: negative (Ct > 38.0).

**Table 5:** Influenza A virus H1 and H3 RT-rtPCR-positive and suspect oral fluids by farm during bi-weekly sample collection with mean positive cycle threshold values and standard error of the mean.

	Farm 1					Farm 2					Farm 3					Farm 4				
	H1		H3		H1/H3	H1		H3		H1/H3	H1		H3		H1/H3	H1		H3		H1/H3
Time	#/# Pos. (%)	Mean Ct (SEM)	#/# Pos. (%)	Mean Ct (SEM)	#/# Pos. (%)	#/# Pos. (%)	Mean Ct (SEM)	#/# Pos. (%)	Mean Ct (SEM)	#/# Pos. (%)	#/# Pos. (%)	Mean Ct (SEM)	#/# Pos. (%)	Mean Ct (SEM)	#/# Pos. (%)	#/# Pos. (%)	Mean Ct (SEM)	#/# Pos. (%)	Mean Ct (SEM)	#/# Pos. (%)
1	-	-	1 *	NA	-	4/8 (50.0) 1 *	34.0 [0.3]	-	-	-	-	-	-	-	-	-	-	-	-	-
2	-	-	-	-	-	1/8 (12.5) 3 *	33.5 [0.0]	-	-	-	1 *	NA	-	-	1 *	1/8 (12.5) 1 *	31.9 [0.0]	-	-	-
3	1 *	NA	-	-	-	2/7 (28.6) 2 *	32.5 [0.3]	-	-	-	-	-	-	-	1 *	-	-	3/49 (6.1)	29.6 [1.2]	-
4	-	-	-	-	-	6/8 (75.0) 1 *	32.2 [0.8]	-	-	-	NT	NT	NT	NT	NT	-	-	-	-	1 *
5	-	-	-	-	-	NT	NT	NT	NT	NT	NT	NT	NT	NT	NT	NT	NT	NT	NT	NT
6	1 *	NA	1 *	NA	-	-	-	-	-	-	-	-	-	-	-	1 *	NA	-	-	-
7	NT	NT	NT	NT	NT	-	-	-	-	-	NT	NT	NT	NT	NT	-	-	1 *	NA	-
8	NT	NT	NT	NT	NT	-	-	-	-	-	-	-	-	-	-	-	-	-	-	-
Farm Total	-	-	-	-	-	13/49 (26.5)	32.9 [0.4]	-	-	-	-	-	-	-	-	1/49 (2.0)	31.9 [0.0]	3/49 (6.1)	30.1 [1.8]	-

H1/H3: number of positive mixed subtypes; #/#: number of H1 or H3 subtypes/total samples tested; ( ): percentage of H1- or H3-positive oral fluids per month; [ ]: standard error of the mean [SEM]; *: samples with suspect Ct values (35.0 < Ct ≤ 38.0); NA: not applicable; NT: none tested at this sample collection time; -: negative (Ct > 38.0).

## Data Availability

The data presented in this study are available upon request from the corresponding authors.
